# An optimal BMI range associated with a lower risk of mortality among HIV-infected adults initiating antiretroviral therapy in Guangxi, China

**DOI:** 10.1038/s41598-019-44279-z

**Published:** 2019-05-24

**Authors:** Junjun Jiang, Xionglin Qin, Huifang Liu, Sirun Meng, Abu S. Abdullah, Jinping Huang, Chunwei Qin, Yanfen Liu, Yunxuan Huang, Fengxiang Qin, Jiegang Huang, Ning Zang, Bingyu Liang, Chuanyi Ning, Yanyan Liao, Hao Liang, Fengyao Wu, Li Ye

**Affiliations:** 1 Guangxi Key Laboratory of AIDS Prevention and Treatment & Guangxi Universities Key Laboratory of Prevention and Control of Highly Prevalent Disease, School of Public Health, Guangxi Medical University, Nanning, Guangxi China; 2 Guigang Center for Disease Control and Prevention, Guigang, Guangxi China; 3 Fourth People’s Hospital of Nanning, Nanning, Guangxi China; 4 Boston University School of Medicine, Boston Medical Center, Boston, Massachusetts 02118 USA; 5 Guangxi Collaborative Innovation Center for Biomedicine, Life Sciences Institute, Guangxi Medical University, Nanning, Guangxi China

**Keywords:** HIV infections, Epidemiology

## Abstract

Previous studies investigating HIV-infected patients suggested a direct link between underweight and the mortality rate of AIDS. However, there was a lack of evidence showing the optimal range of initial body mass index (BMI) patients maintain during antiretroviral therapy (ART). We aimed to evaluate associations of the BMI values pre-ART and during the entire ART duration with mortality among HIV-positive individuals. In total, 5101 HIV/AIDS patients, including 1439 (28.2%) underweight, 3047 (59.7%) normal-weight, 548 (10.7%) overweight and 67 (1.3%) obese patients, were included in this cohort. The cumulative mortality of underweight, normal-weight, and overweight were 2.4/100 person-years (95% *CI* 1.9–2.9), 1.1/100 person-years (95% *CI* 0.9–1.3), and 0.5/100 person-years (95% *CI* 0.1–0.9), respectively. Cumulative mortality was lower in both the normal-weight and overweight populations than in the underweight population, with an adjusted hazard ratio (*AHR*) of 0.5 (95% *CI* 0.4–0.7, *p* < 0.001) and 0.3 (95% *CI* 0.1–0.6, *p* = 0.002), respectively. Additionally, in the 1176 patients with available viral load data, there was significant difference between the underweight and normal-weight groups after adjustment for all factors, including viral load (*p* = 0.031). This result suggests that HIV-infected patients in Guangxi maintaining a BMI of 19–28 kg/m^2^, especially 24–28 kg/m^2^, have a reduced risk of death.

## Introduction

Since the human immunodeficiency virus (HIV)/acquired immunodeficiency syndrome (AIDS) epidemic crisis in 1981, HIV/AIDS has been one of the leading causes of death worldwide^1^. As of December 31, 2017, there were 985,034 reported cases of HIV/AIDS in China, with 232,290 reported death, resulting in a mortality rate of 23.6%^2^. Guangxi, the province with the second-highest HIV prevalence rate in Southwest China, according to the report of the Guangxi Health and Family Planning Commission^3^, had approximately 118 thousand total HIV/AIDS patients as of January 31, 2017, with 40,500 deaths, resulting in a mortality rate (34.3%) higher than the national average (23.8%) during the same period. Currently, the most frequently reported risk factors for HIV-related mortality in people living with HIV/AIDS (PLWHA) were not receiving antiretroviral therapy (ART)^4,5^ and having delayed ART initiation^6^. Clearly, due to the implementation of ART, the morbidity and mortality rates in PLWHA have significantly decreased^7,8^. Globally, an estimated 18.2 million PLWHA had received ART as of mid-2016, approximately 1.1 million patients had died of AIDS-related causes at the end of 2015, and the AIDS-related mortality rate decreased by approximately 28% from 2000 to 2015^9^.

PLWHA on ART remain at higher risk of death than the general population^10^. In China, the National Free Antiretroviral Treatment Program (NFATP), initiated in 2002, and scaled up in 2003, has successfully reduced the overall HIV mortality rate^5^. However, in spite of the expansion of antiviral treatment coverage, China is still facing an enormous challenge from the high rate of AIDS-related death^11^. Wang *et al*. examined the temporal trend in HIV/AIDS-related deaths in China and found that the mortality rate increased significantly from 2000–2012, with an average annual percentage change of 22.3%^12^.

The average mortality rate among all treated patients was 2.63/100 person-years in Guangxi, China from 2010 to 2015^13^. Studies have reported many independent factors related to death among ART patients, including late diagnosis^14^, advanced WHO disease stage^15–17^ and lower CD4 cell count^5,15,17,18^. In addition to these factors, many studies have shown that a low body mass index (BMI), mostly a BMI of <18.5 kg/m^2^, seems to be a risk factor for mortality in PLWHA on ART^15,17,19–21^.

Historically, studies have used BMI as the general obesity index. The BMI classification is defined in China as follows^22,23^: underweight (<18.5 kg/m^2^), normal weight (18.5–23.9 kg/m^2^), overweight (24–27.9 kg/m^2^), and obese (≥28 kg/m^2^). The full spectrum of weight categories is seen in HIV patients currently, and different levels of BMI seem to be closely related to AIDS progression in PLWHA. A longitudinal study of the Miami HIV-1-infected drug abusers cohort suggests that mild-to-moderate obesity in HIV-1-infected chronic drug users does not impair immune function and is associated with improved HIV-1-related survival rates^24^. A low baseline BMI and a decreasing BMI during follow-up were independently predictive of progression to AIDS, meaning that overweight patients may progress more slowly to AIDS^25^. In addition, many other studies similarly reported that a low baseline BMI and a BMI decrease were associated with a high risk of HIV progression and opportunistic infections during follow-up^26–29^. However, in Kim *et al*.’s study, overweight and obese HIV patients had a higher risk of multimorbidity than normal or underweight patients^30^. In another study, weight gain among underweight or normal-weight HIV ART participants predicted reduced inflammation and improved survival, but this effect did not apply to overweight or obese participants^31^.

Zhang *et al*. conducted a survival analysis of PLWHA on ART in Ningbo, China and found that a BMI of <18.5 was one of the risk factors for mortality^19^. Two studies in Guangxi showed that a BMI of <18 kg/m^2^ was associated with an increased risk of asymptomatic pulmonary tuberculosis among HIV patients^32^ and that HIV patients with an initial BMI of <18.5 kg/m^2^ had increased mortality^33^. In this study, we retrospectively collected data from China’s National Free Antiretroviral Treatment Program (NFATP) in Nanning city and Guigang city in Guangxi, China, aiming to ascertain the BMI variance status and to investigate the association between BMI and mortality in Chinese HIV patients on ART.

## Results

### Baseline characteristics

**Figure 1:**
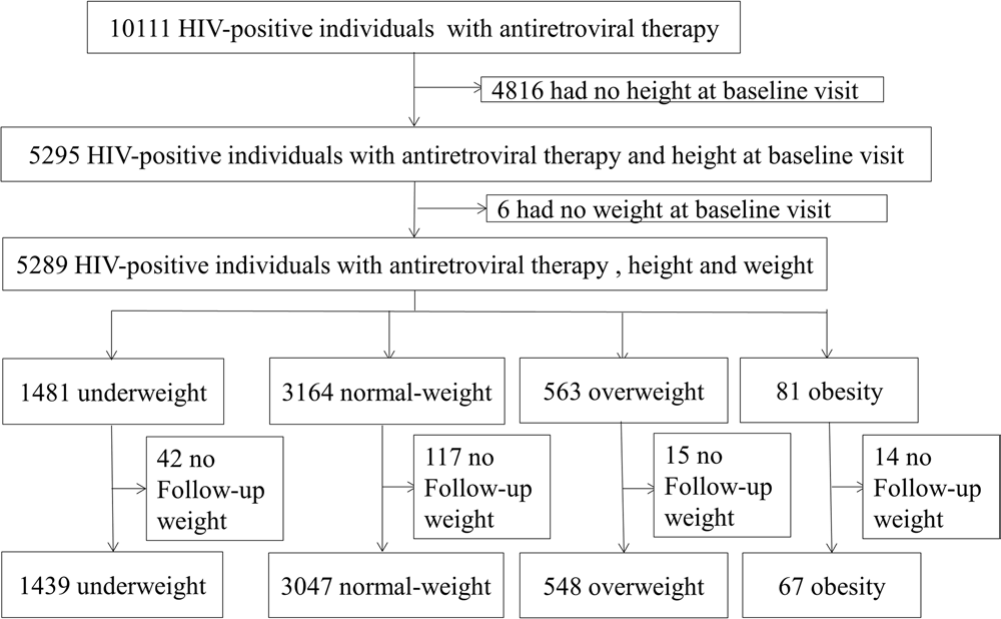
Chart of the inclusion and exclusion criteria in this study. A total of 10,111 HIV-positive patients received combination ART in the study settings, but 4816 had no height data and 6 had no weight data at baseline, and 190 had no weight data during follow-up; these patients were excluded, leaving a total of 5,101 individuals enrolled in this study.

Data were collected from May 19, 2005 to June 30, 2016. A total of 10,111 HIV-positive patients received combination ART in the study settings. Of these patients, we excluded 4816 without a recorded height, 6 without recorded baseline weight, and 190 without recorded follow-up weight, leaving 5101 individuals enrolled (12,458.9 person-years) (Fig. 1). Among these patients, 1439 (28.2%) were underweight, 3047 (59.7%) had a normal weight, 548 (10.7%) patients were overweight and 67 (1.3%) were obese. Table 1 shows the baseline characteristics of the participants. Among the participants, 1281 (25.1%) patients were aged 30–39 years; the mean age of the patients was 46.86 years (32.79–60.93), and the median age was 45 years (IQR 36–58). Among the eligible patients, 3538 (69.4%) were male, and 3524 (69.1%) were married or living with a partner. Most (63.2%) patients’ CD4 T cell count were less than 200 cells/μL. The main route of HIV infection was heterosexual intercourse (85.1%), and 1286 (25.2%) patients’ initial antiretroviral regimen was EFV + 3TC + TDF (Efavirenz, Lamivudine, Tenofovir disoproxil fumarate). A total of 1903 (37.3%) patients were in WHO clinical stage I before ART. The four groups were significantly different in age, sex, marital status, CD4 T cell count, route of HIV infection, initial antiretroviral regimen and WHO clinical stage before ART. The median CD4 T cell count was 51 (20–172) cells/μL in the underweight group, 149 (41–274) cells/μL in the normal-weight group, 221(106–322) cells/μL in the overweight group and 281 (200–368) cells/μL in the obese group.

**Table 1:** Characteristics of patients with HIV infection at ART initiation.

Variable	Total	Baseline BMI < 18.5	Baseline 18.5 ≤ BMI < 24	Baseline 24 ≤ BMI < 28	Baseline BMI ≥ 28	*χ* ^2^ */F*	*p*
Total	5101	1439	3047	548	67	—	—
Age						39.83	<0.001
<30	561 (11.0%)	189 (13.1%)	322 (10.6%)	44 (8.0%)	6 (9.0%)		
30–39	1281 (25.1%)	377 (26.2%)	758 (24.9%)	125 (22.8%)	21 (31.3%)		
40–49	1248 (24.47%)	320 (22.2%)	785 (25.8%)	127 (23.2%)	16 (23.9%)		
50–59	894 (17.53%)	221 (15.4%)	528 (17.3%)	134 (24.5%)	11 (16.4%)		
≥60	1117 (21.9%)	332 (23.1%)	654 (21.5%)	118 (21.5%)	13 (19.4%)		
Gender						19.68	<0.001
Male	3538 (69.4%)	946 (65.7%)	2184 (71.7%)	366 (66.8%)	42 (62.7%)		
Female	1563 (30.6%)	493 (34.3%)	863 (28.3%)	182 (33.2%)	25 (37.3%)		
Marital status						49.79	<0.001
Married or living with a partner	3524 (69.1%)	904 (62.8%)	2143 (70.3%)	427 (77.9%)	50 (74.6%)		
Single, divorced or widowed	1566 (30.7%)	532 (37.0%)	897 (29.4%)	120 (21.9%)	17 (25.4%)		
Unknown	11 (0.2%)	3 (0.2%)	7 (0.2%)	1 (0.2%)	0 (0%)		
CD4 cell count at hospital admission (cells/µL)	126 (33–265)	51 (20–172)	149 (41–274)	221 (106–322)	281 (200–368)	—	<0.001
<100	2292 (44.9%)	920 (63.9%)	1233 (60.2%)	131 (23.9%)	8 (11.9%)		
100–199	932 (18.3%)	211 (14.7%)	602 (19.8%)	110 (20.1%)	9 (13.4%)		
200–349	1304 (25.6%)	225 (15.6%)	857 (28.1%)	194 (35.4%)	28 (41.8%)		
≥350	550 (10.8%)	78 (5.4%)	341 (11.2%)	109 (19.9%)	22 (32.8%)		
Missing	23 (0.5%)	5 (0.4%)	14 (0.5%)	4 (0.7%)	0 (0%)		
Viral load (median, IQR)	126396 (38168, 357323)	186208 (62650, 459865)	110946 (32943, 327062)	74050 (20454, 186234)	26918 (14196, 83038)	2.60	0.049
Route of HIV infection							
Blood or plasma transfusion	15 (0.3%)	4 (0.3%)	10 (0.3%)	0 (0%)	1 (1.5%)	8.29	0.762
Homosexual intercourse	269 (5.3%)	76 (5.3%)	162 (5.3%)	26 (4.7%)	5 (7.5%)		
Heterosexual intercourse	4343 (85.1%)	1217 (84.6%)	2599 (85.3%)	474 (86.5%)	53 (79.1%)		
Intravenous drug use	356 (7.0%)	108 (7.5%)	208 (6.8%)	34 (6.2%)	6 (9.0%)		
Other or unknown	118 (2.3%)	34 (2.4%)	68 (2.2%)	14 (2.6%)	2 (3.0%)		
Initial antiretroviral regimen						121.90	<0.001
EFV + 3TC + TDF	1286 (25.2%)	382 (26.6%)	755 (24.8%)	130 (23.7%)	19 (28.4%)		
EFV + 3TC + AZT	717 (14.1%)	172 (12.0%)	449 (14.7%)	83 (15.2%)	13 (19.4%)		
EFV + 3TC + D4T	628 (12.3%)	259 (18.0%)	336 (11.0%)	31 (5.7%)	2 (3.0%)		
NVP + 3TC + AZT	566 (11.1%)	90 (6.3%)	388 (12.7%)	78 (14.2%)	10 (14.9%)		
Other or unknown	1904 (37.3%)	536 (37.3%)	1119 (36.7%)	226 (41.2%)	23 (34.3%)		
WHO clinical stage before ART						268.71	<0.001
1	1903 (37.3%)	330 (22.9%)	1239 (40.7%)	292 (53.3%)	42 (62.7%)		
2	474 (9.3%)	115 (8.0%)	291 (9.6%)	63 (11.5%)	5 (7.5%)		
3	908 (17.8%)	302 (21.0%)	522 (17.1%)	72 (13.1%)	12 (17.9%)		
4	1816 (35.6%)	692 (48.1%)	995 (32.7%)	121 (22.1%)	8 (11.9%)		

The data are the medians (IQRs) or n (%). Age, gender, marital status, CD4 T cell count, viral load regimen, and WHO clinical stage before ART were significantly associated in different BMI groups (p < 0.05). AZT = zidovudine. 3TC = lamivudine. NVP = nevirapine. EFV = efavirenz. D4T = stavudine. TDF = tenofovir.

### The mortality rate of HIV-infected patients who received ART in the different BMI groups

**Figure 2:**
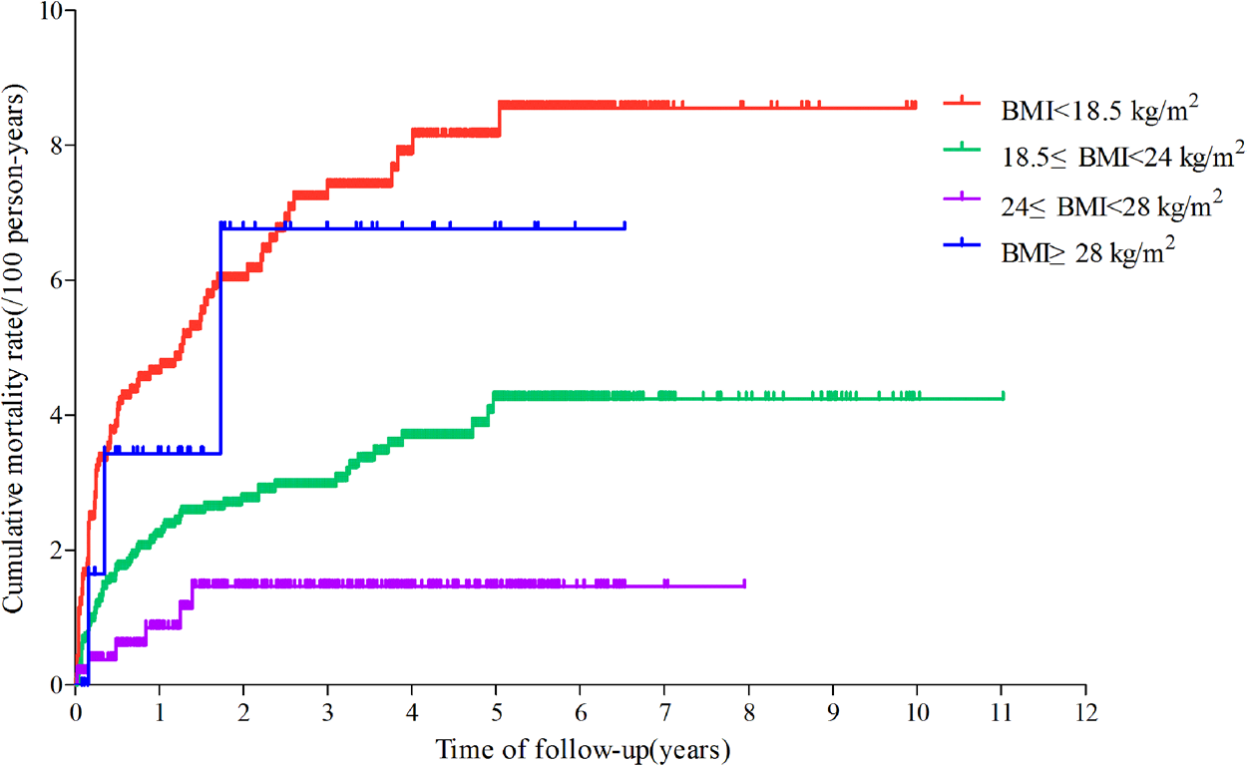
Cumulative mortality rates of patients in the different BMI groups. Figure 2 shows that the cumulative mortality rate in the underweight group was significantly higher than that in the overweight and normal-weight groups and that the mortality rate in the obese group was higher than that in the normal-weight and overweight groups.

The mortality rate was 1.4/100 person-years (95% *CI* 1.2–1.6) in Guangxi. The mortality rates in the four groups were significantly different (*p* < 0.001). The mortality rate was 2.4/100 person-years (95% *CI* 1.9–2.9) in the underweight group, 1.1/100 person-years (95% *CI* 0.9–1.3) in the normal-weight group, 0.5/100 person-years (95% *CI* 0.1–0.9) in the overweight group and 2.4/100 person-years (95% *CI* −0.2–4.9) in the obese group (Table 2). Figure 2 shows that the cumulative mortality rate in the underweight group was significantly higher than that in the overweight and normal-weight groups and that the mortality rate in the obese group was higher than that in the normal-weight and overweight groups.

**Table 2:** Mortality rates of HIV-infected patients who started ART.

Baseline BMI	HIV-infected patients With ART, No.	Deaths, No.	Person-years	Deaths/100 Person-Years (95% *CI*)	*χ* ^2^	*p*
					39.34	<0.001
BMI < 18.5	1439	85	3493.1	2.4 (1.9–2.9)		
18.5 ≤ BMI < 24	3047	83	7595.2	1.1 (0.9–1.3)		
24 ≤ BMI < 28	548	6	1243.6	0.5 (0.1–0.9)		
BMI ≥ 28	67	3	127.9	2.4 (−0.2–4.9)

### Associations between BMI and time to death

We used a Cox proportional hazards regression model to control for confounding factors. Table 3 shows that patients in the normal-weight group had a lower risk of death than those in the underweight group, while patients in the overweight group had the lowest risk of death. With respect to the underweight group, the hazard ratio of overweight adjusted for all factors except viral load was 0.3 (95% *CI* 0.1–0.6, *p* = 0.002). In addition, there was a significant difference in the mortality rate (*p* < 0.001) between the underweight and normal-weight groups when adjusted for all factors except viral load. There was also a significant difference between the underweight and normal-weight groups after adjustment for all factors, including viral load (*p* = 0.031).

**Table 3:** Associations between BMI and time to death.

	BMI < 18.5	18.5 ≤ BMI < 24	24 ≤ BMI < 28	BMI ≥ 28
Number of events	1439	3047	548	67
Time on antiretroviral treatment (person-years)	3493.1	7595.2	1243.6	127.9
*AHR*^a^ (95% *CI*), *p* value	—	0.5 (0.4–0.7), <0.001	0.3 (0.1–0.6), 0.002	1.3 (0.4–4.2), 0.705
Number of patients who have virus load records	354	693	117	12
Time on antiretroviral treatment (person-years)	1450.3	2821.6	456.8	45.9
*AHR*^b^ (95% *CI*), *p* value	—	0.5 (0.3–0.9), 0.031	—	3.2 (0.6–17.3), 0.187

*AHR*^a^: adjusted by age, gender, marital status, baseline BMI, baseline CD4 T cell count, and clinical disease (including TB infection, Skin lesion, thrush, oral hairy leukoplakia, persistent diarrhoea, continuous or intermittent fever, recurrent severe bacterial infections, disseminated non-tuberculosis bacillus infection, oesophageal candidiasis, extrapulmonary cryptococcal infection, *Yersinia pneumocystis* pneumonia, disseminated fungal disease, cytomegalovirus infection, extrapulmonary tuberculosis, repeated severe bacterial pneumonia, chronic herpes simplex virus infection, herpes zoster, Toxoplasma encephalopathy, brain lymphoma, and WHO clinical stage).

*AHR*^b^: adjusted by the viral load on the basis of the *AHR*^*a*^.

### Stratified analysis

We further controlled for mixed factors by stratified analysis. The mortality rates were significantly different among the underweight, overweight and obese groups, as stratified by the baseline characteristics of age (30–39 and ≥60), sex (male), marital status (married or living with a partner), WHO clinical stage before ART (stages 1, 2 and 3), CD4 T cell count (<200 cells/μL) and initial antiretroviral regimen (EFV + 3TC + TDF). There was no significant difference in other baseline characteristics (Supplementary Table S1).

**Figure 3:**
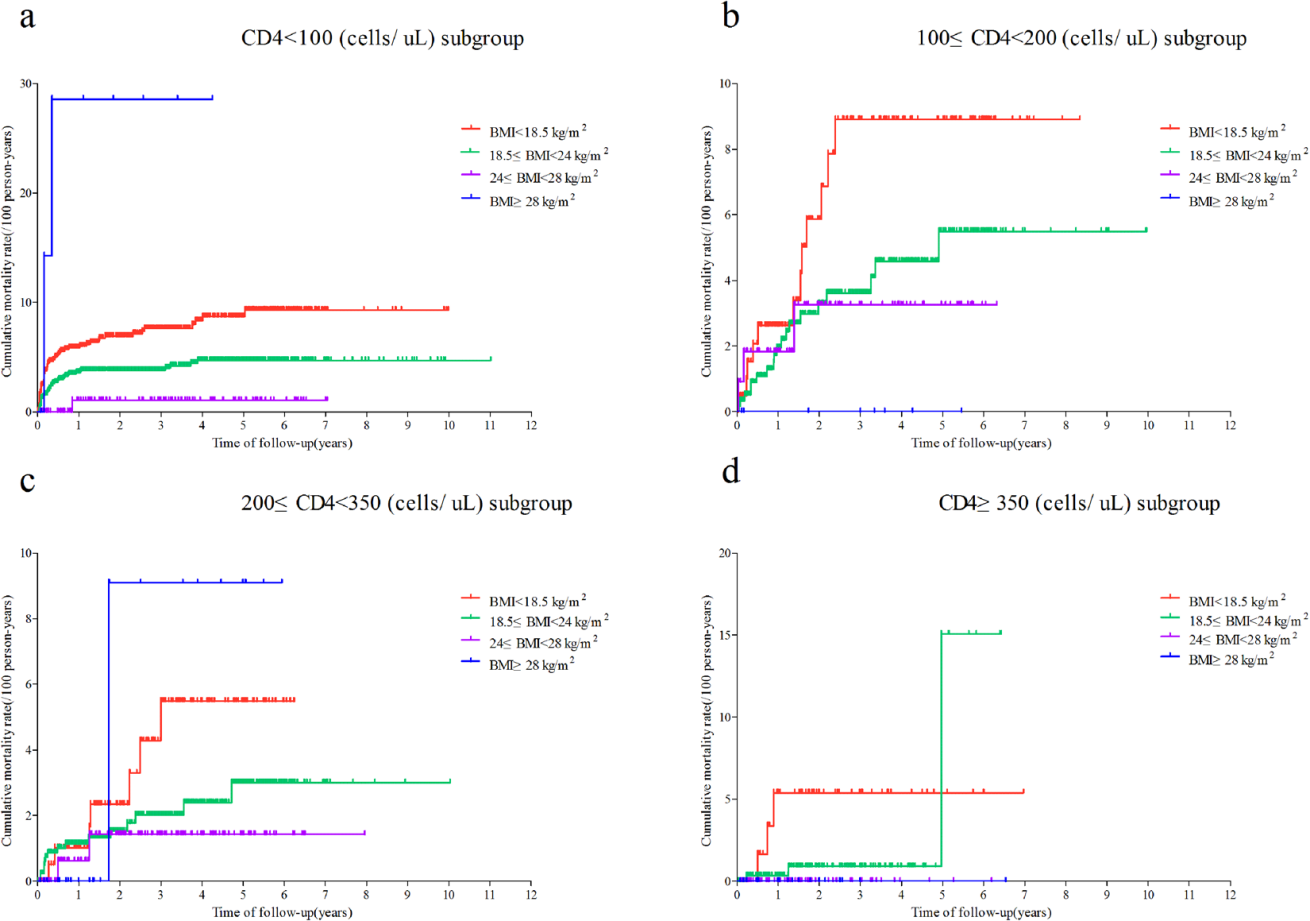
Kaplan-Meier Survival Curves for the four weight groups in each stratum of CD4 T cell count. (**a**) Cumulative mortality rate of HIV-infected patients in the “CD4 < 100/µL” subgroup. (**b**) Cumulative mortality rate of HIV-infected patients in the “100 ≤ CD4 < 200/µL” subgroup. (**c**) Cumulative mortality rate of HIV-infected patients in the “200 ≤ CD4 < 350/µL” subgroup. (**d**) Cumulative mortality rate of HIV-infected patients in the “CD4 ≥ 350/µL” subgroup.

We further analysed the cumulative mortality rates in the four groups stratified into different CD4 T cell count groups. Figure 3 shows that there was a significant difference in the Kaplan-Meier analysis results between the CD4 < 100 cells/μL group and the other three CD4 groups. In the CD4 < 100 cells/μL group, the cumulative mortality rate was the highest in the underweight group and was higher in the BMI < 18.5 kg/m^2^ group than in the normal-weight and obese groups; the overweight group had the lowest mortality rate, which was similar to that in the group with 200 < CD4 < 350 cells/μL. In the 100 < CD4 < 200 cells/μL group, the cumulative mortality was inversely related to the BMI with respect to the time of follow-up (years). In the CD4 > 350 cells/μL group, the mortality rates in the underweight and normal-weight groups were higher than those in the other two groups. We observed the effect of BMI on the mortality rate in the different CD4 groups: patients with a lower BMI had a higher risk of mortality when the CD4 cell count was less than 350cells/μL, similar to the results without stratification by the CD4 T cell count.

### BMI changes during ART and mortality rate changes by BMI

**Figure 4:**
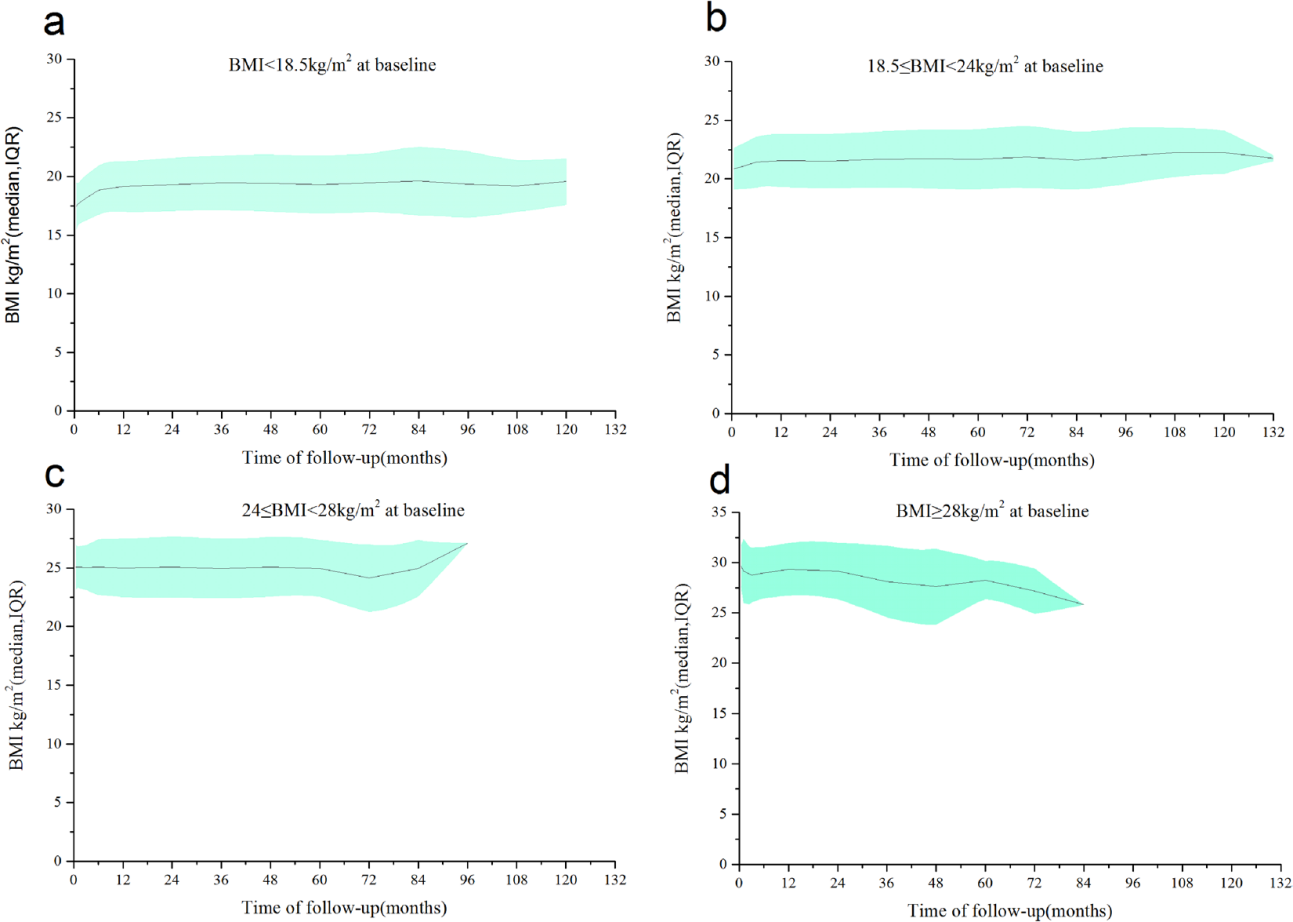
Mean BMI and standard deviation with respect to the time of follow-up in the four baseline BMI groups. (**a**) Average BMI with respect to follow-up time in the underweight group. (**b**) Average BMI with respect to follow-up time in the normal-weight group. (**c**) Average BMI with respect to follow-up time in the overweight group. (**d**) Average BMI with respect to follow-up time in the obese group.

**Figure 5:**
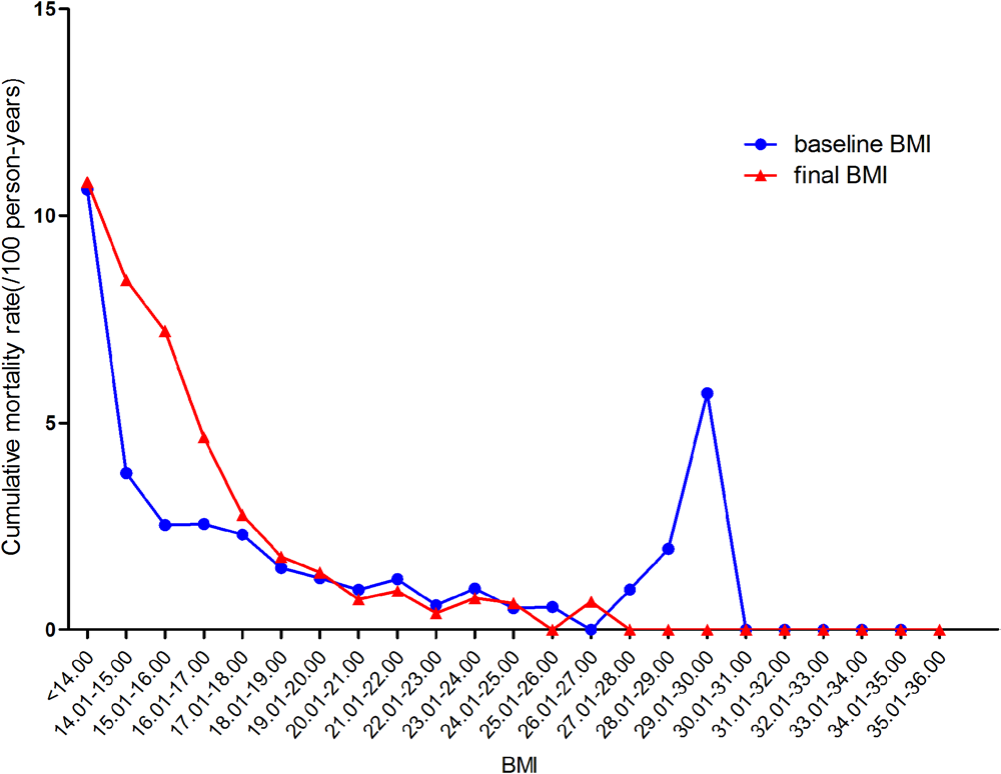
Changes in the cumulative mortality rates of patients with respect to baseline BMI and final BMI.

Figure 4 shows the average BMI with respect to the time of follow-up in the four baseline BMI groups. Among the different BMI groups, there was almost no change in the patients’ BMI values from baseline to the end of follow-up. Figure 5 indicates the change in the mortality rate with respect to BMI; the mortality rate decreased with increases in the baseline and final BMI. This figure demonstrates that the mortality rate was relatively lower for a BMI of 19–28 kg/m^2^ and was lowest for a BMI of 24–28 kg/m^2^.

## Discussion

This study was a retrospective cohort study over an 11-year period (2005–2016) in HIV-infected patients who received ART in Guangxi, China. The mortality rate was 1.4/100 person-years (95% *CI* 1.2–1.6). In an earlier study in Guangxi, the average mortality rate among all treated patients was 2.6/100 person-years from 2010 to 2015^13^. In our study, the mortality rate of HIV-infected patients who received ART in the different BMI groups was 1.42/100 person-years. Possible reasons for the difference in the mortality rate between our study and that of Tang *et al*.^13^ include: (1) The earlier study^13^ included HIV patients from all cities of Guangxi, including the two cities that we studied in the current study. (2) The eligible patients in our study were PLWHA with ART who had available BMI data. Therefore, the characteristics of the subjects were different from those in the earlier study. In a meta-analysis conducted in resource-limited countries of Africa, Asia, and South and Central America, mortality rates ranged from 2.6% to 29.7%^34^, higher than those observed in our study. The highest rate was reported in a multi-regional South American cohort and the lowest was in sub-Saharan Africa^34^.

In our study, higher baseline BMI (19–28 kg/m^2^) was associated with a lower mortality rate than that in the underweight group. We compared the baseline characteristics among the four groups and found significant differences among these four groups; in addition, we found that the patients in the normal and overweight groups had a lower risk of death than those in the underweight group (the hazard ratios adjusted for all factors were 0.5 (95% *CI* 0.4–0.7) and 0.3 (95% *CI* 0.1–0.6) for normal-weight and overweight, respectively). When we controlled for the baseline characteristics by stratified analysis, we found similar results—the mortality rate in the normal-weight group was lower than that in the underweight and overweight groups. Similarly, Troiano *et al*.^35^ showed an U-shaped association between BMI and mortality. Studies in Southeast Asia^36,37^ and sub-Saharan Africa^38–40^ reported that a low baseline BMI predicts early mortality in resource-limited settings. In another study, the mortality rate of underweight PLWHA prior to ART was almost two-fold that of normal-weight PLWHA in the 1st two years of ART^41^. A meta-analysis showed that compared to normal-weight, overweight was related to lower all-cause mortality, while the outcome was opposite for obesity^42^. We also found that BMI changed little from baseline to the end of follow-up, which further demonstrated that the baseline BMI and mortality rate are closely related.

In our study, the CD4 T cell count was a key differential factor among the four groups. Therefore, we controlled for the CD4 T cell count via stratified analysis. We found that the mortality rate in the underweight group was higher than that in the normal-weight group, which was higher than that in the overweight group, for patients with CD4 < 350 cells/μL but was slightly different for patients with CD4 > 350 cells/μL; the reason may be the small sample size, indicating that in the different CD4 T cell count groups, the effect of BMI on the mortality rate of patients in this study is generally similar. Regarding the change in the CD4 T cell count during ART, our findings are consistent with other findings that showed an enhanced increase in the CD4 T cell count of 1 year after ART initiation^43^. Similarly, in other study, there was no difference associated with weight in the undetectable viral load or the increase in the CD4 T cell count at 3–9 months after ART initiation^44^. Therefore, we suggest that underweight HIV patients in Guangxi who received ART may be at greater risk of mortality and that patients maintaining a BMI of 19–28 kg/m^2^, especially 24–28 kg/m^2^, have a relatively lower risk of mortality. However, a study showed that weight may affect immune cell counts over the course of ART^45^. In other studies, increased BMI values were associated with increased CD4 counts and improved survival^24,25^. Similarly, several previous studies have shown that increases in BMI were positively associated with increased CD4+ T cell counts in HIV-negative children and women^46–48^.

Adipose tissue is an active endocrine and paracrine organ that regulates energy storage, immunity and inflammation^48^. Adipocytes secrete cytokines and adipokines, including adiponectin and leptin. The plasma leptin concentration is positively related to the percentage of adipose tissue^49^. Leptin is a hormone that is similar to cytokines in both function and structure^50^. Both thymic development and peripheral lymphocyte count and function are reduced in leptin-deficient mice^51^. In some studies, leptin replacement therapy reversed the defects in lymphocyte numbers and the decrease in function in humans^52,53^. Therefore, adipokines may be the possible mechanism underlying the effect of BMI on mortality in HIV-infected patients.

There were a few limitations in this study. First, this study is a retrospective cohort study, so there may be bias in subject selection, leading to a significant difference in the baseline characteristics. However, we controlled for and adjusted the factors by stratified analysis and multivariate analysis. Second, due to the small number of obese patients in the study, the results for the obese group should be considered with caution. Third, most of the viral load data were missing, and we were not able to assess the real influence of the viral load on the results. However, despite these limitations, the study provides meaningful epidemiological data on decreasing the mortality risk and prolonging the survival time of HIV-infected patients who receive ART. Additionally, to our knowledge, this study is the first retrospective cohort study over an 11-year period in China—even in Asia—and has great public health significance.

In conclusion, compared to normal weight, underweight increased the risk of death among PLWHA who received ART, while overweight decreased the risk of death. Our study provides further evidence that underweight may contribute to increased mortality in HIV-positive patients and suggests the optimal range of BMI in PLWHA in Guangxi, China.

## Methods

### Study design and included patients

This HIV treatment retrospective cohort study was conducted in Guigang and Nanning cities, Guangxi, China. PLWHA who received antiretroviral treatment (ART) were included. Beginning in 2002, the criteria to receive free combination ART were as follows: (1) a CD4 T cell count of <200 cells/μL, (2) a total lymphocyte count of less than 1200 cells/μL, or (3) a WHO disease stage of 3 or 4^22^. In 2008, the criteria were changed to the following: (1) a CD4 count of ≤350 cells/μL or a WHO disease stage of 3 or 4^23^. The inclusion criteria for this study were as follows: PLWHA ≥18 years of age who received standard ART and had baseline height and weight data and follow-up weight data. The exclusion criteria were as follows: absence of baseline weight or height data or of follow-up weight data, presence of severe liver or kidney disease, and pregnancy (in women). All included patients had weight records, and most had records of the CD4 T cell count, viral load clinical index, other diseases, ART regimen and survival status at fixed time points (0.5, 1, 2, and 3 months after initiation of ART and followed up thereafter every three months). However, if the treatment programme was changed, the follow-up period was restarted. Pre-ART BMI data was not changed even if the treatment programme changed.

### Ethics statement

In this study, every patient provided written informed consent for our specific retrospective study before enrolment. The study procedures were clearly explained to the participants, and they were offered the opportunity to ask questions. All methods were performed in accordance with the relevant guidelines and regulations, and all methods were approved by the Ethics and Human Subjects Committee of Guangxi Medical University (Ethical Review No. 20130305-17).

### Data collection and definitions

Data were collected from the Fourth People’s Hospital of Nanning city and from the Guigang Center for Disease Control and Prevention (CDC). These data were collected from May 19, 2005 to June 30, 2016. In the study, baseline information was collected at initiation of ART. BMI was calculated according to the following formula: “BMI = weight (kg)/height (m)^2^” and the grade of BMI was calculated following the criteria established by the Health Industry Standards of the People’s Republic of China: underweight (BMI < 18.5 kg/m^2^), normal weight (18.5–24.0 kg/m^2^), overweight (24.0–28.0 kg/m^2^), and obese (>28.0 kg/m^2^)^22,23^. The CD4 T cell count stage was classified as follows: stage 1 (≥350cells/μL), stage 2 (200–350 cells/μL), stage 3 (200–349 cells/μL), and stage 4 (<200cells/μL). We collected baseline and follow-up weight records to calculate BMI values. The outcomes included censored data and death. June 30, 2016 was established as the last date of follow-up.

### Definition of clinical diseases and clinical test indexes

All clinical diseases includes the following: TB infection, skin lesion, thrush, oral hairy leukoplakia, persistent diarrhoea, continuous or intermittent fever, recurrent severe bacterial infections, disseminated non-tuberculosis bacillus infection, oesophageal candidiasis, extrapulmonary cryptococcal infection, *Yersinia pneumocystis* pneumonia, disseminated fungal disease, cytomegalovirus infection, extrapulmonary tuberculosis, repeated severe bacterial pneumonia, chronic herpes simplex virus infection, herpes zoster, toxoplasma encephalopathy, Kaposi sarcoma, and brain lymphoma. Clinical test indexes included white blood cell count, lymphocyte count, blood platelet count, haemoglobin, serum creatinine, blood urea nitrogen, triglyceride, total cholesterol, blood glucose, blood amylase, aspartate aminotransferase, alanine aminotransferase and total bilirubin.

### Statistical analysis

The mortality of HIV-positive patients was calculated by the number of annual deaths divided by the total number of 100 person-years. We used a Chi-square test (for categorical variables) to compare the characteristics among the four BMI groups (underweight, normal-weight, overweight, and obese). Kaplan-Meier analysis was used to calculate survival probabilities for cumulative mortality, and Cox proportional hazards ratios were used to evaluate the related factors among the four groups. We compared the four groups using Cox proportional hazards regression analyses stratified by age, sex, CD4 T cell count, marital status, WHO clinical stage before ART, route of HIV infection, and initial antiretroviral regimen to estimate hazard ratios (*H*Rs; reported with 95% *CI*s) and adjusted hazard ratios (*AHRs*; reported with 95% *CI*s). To ensure that the characteristics did not affect the results among the four groups, we used a Chi-square test for each baseline characteristic and clinical disease; we then used stratified analysis to control for and balance the baseline characteristics between the normal-weight and overweight groups and Cox proportional hazards models to evaluate and adjust the mortality results among the underweight, overweight, and obese groups. The data were analysed using Statistical Package for the Social Sciences (SPSS) version 20.0 (SPSS Inc., Chicago, USA) and GraphPad Prism version 6.0 (GraphPad Software, San Diego, California, USA).

### Ethical statement

In this study, every patient provided written informed consent for our specific retrospective study before enrolment. The study procedures were clearly explained to the participants, and they were offered the opportunity to ask questions. The study was approved by the Ethics and Human Subjects Committee of Guangxi Medical University (Ethical Review No. 20130305-17).

## Supplementary information

Supplementary Table S1

## Data Availability

Since the data contain information of HIV-infected patients, which involve patient privacy, they cannot be disclosed.
