# Supplementary material for: An optimal BMI range associated with a lower risk of mortality among HIV-infected adults initiating antiretroviral therapy in Guangxi, China
Source: Sci Rep. 2019 May 24;9:7816. doi: 10.1038/s41598-019-44279-z (PMC6534550; doi:10.1038/s41598-019-44279-z)
Supplement: Supplementary file 1 — Supplementary Table S1 [file 41598_2019_44279_MOESM1_ESM.docx]

**An optimal BMI range associated with a lower risk of mortality among HIV-infected adults initiating antiretroviral therapy in Guangxi, China**

Junjun Jiang^1#^, Xionglin Qin^2#^, Huifang Liu^1#^, Sirun Meng^3^, Abu S Abdullah^4^, Jinping Huang^3^, Chunwei Qin^2^, Yanfen Liu^3^, Yunxuan Huang^2^, Fengxiang Qin^1^, Jiegang Huang^1^, Ning Zang^5^, Bingyu Liang^1^，Chuanyi Ning^5^, Yanyan Liao^5^ ,Hao Liang^1,5*^, Fengyao Wu^3*^, Li Ye^1*^

| Supplementary Table S1. Stratified analysis of general characteristics for effects of different baseline BMI on mortality of HIV/AIDS patients with ART. | | | | | | |
| --- | --- | --- | --- | --- | --- | --- |
| Stratified variable | 18.5≤ BMI<24 vs BMI<18.5 | | 24≤BMI<28 vs BMI<18.5 | | BMI≥28 vs BMI<18.5 | |
|  | *AHR**(95%CI) | *p* | *AHR**(95%*CI*) | *p* | *AHR**(95%CI) | *p* |
| **Age** |  |  |  |  |  |  |
| <30 | 1.0(0.2-4.8) | 0.955 | 1.0(0.1-17.5) | 0.981 | 1.6 (0.0-439.9) | 0.868 |
| 30-39 | 0.4(0.2-0.8) | 0.016 | - |  | - |  |
| 40-49 | 0.5 (0.2-1.2) | 0.143 | 0.3(0.0-2.4) | 0.253 | - | 0.995 |
| 50-59 | 0.3(0.1-1.5) | 0.131 | 0.2(0.0-2.2) | 0.182 | 0.1 (0.0-3615441.6) | 0.805 |
| >=60 | 0.5 (0.3-0.9) | 0.031 | 0.4(0.1-1.4) | 0.158 | 1.4 (0.3-7.8) | 0.676 |
| **Sex** |  |  |  |  |  |  |
| Male | 0.5(0.3-0.7) | <0.001 | 0.2 (0.1-0.6) | 0.004 | 1.6(0.5-5.3) | 0.476 |
| Female | 0.6 (0.3-1.4) | 0.24 | 0.4 (0.1-3.7) | 0.458 | - | 0.984 |
| **Marital status** |  |  |  |  |  |  |
| Married or living with partner | 0.4 (0.3-0.6) | <0.001 | 0.3(0.1-0.7) | 0.004 | 1.8(0.5-0.7) | 0.353 |
| Single,divorced or widowed | 0.8 (0.4-1.7) | 0.655 | - | 0.902 | - | 0.914 |
| Unknown | - | 0.992 | - | 0.999 | - |  |
| **WHO clinical stage before ART** |  |  |  |  |  |  |
| I | 0.2(0.1-0.5) | <0.001 | 0.2 (0.0-1.0) | 0.056 | 0.2(0.0-3.9) | 0.265 |
| II | 0.248(0.076-0.813) | 0.021 | 0.1 (0.0-1.7) | 0.12 | - | 0.615 |
| III | 0.4 (0.2-0.8) | 0.011 | 0.2 (0.0-1.3) | 0.102 | - | 0.972 |
| IV | 0.8 (0.5-1.4) | 0.435 | 0.2 (0.0-1.6) | 0.132 | 26.6 (5.1-138.9) | <0.001 |
| **CD4 cell count (cells per ul)** |  |  |  |  |  |  |
| <100 | 0.5(0.3-0.8) | 0.001 | 0.1(0.0-0.9) | 0.035 | 5.5(1.2-25.3) | 0.030 |
| 100-199 | 0.3 (0.1-0.8) | 0.014 | 0.5(0.1-2.3) | 0.345 | - | - |
| 200-349 | 1.0 (0.3-3.1) | 1 | 1.0 (0.2-4.5) | 1 | 1.0 (0.0-24.0) | 1 |
| >=350 | - | 0.427 | - | 0.241 | - | 0.381 |
| Missing | - | 1 | - | 1 | - | - |
| **Initial antiretroviral regimen** |  |  |  |  |  |  |
| EFV+3TC+TDF | 0.3(0.1-0.8) | 0.018 | - | 0.979 | - | 0.996 |
| EFV+3TC+AZT | 0.2 (0.0-28.6) | 0.552 | 0.1 (0.0-403199.9) | 0.788 | 99.9(0.0-242051.8) | 0.247 |
| EFV+3TC+D4T | 0.7 (0.3-1.8) | 0.496 | - | 0.989 | 13.3 (0.9-206.1) | 0.640 |
| NVP+3TC+AZT | 0.6 (0.1-5.0) | 0.645 | - | 0.937 | - | 0.985 |
| Other or unknown | 0.5(0.3-0.7) | 0.001 | 0.5(0.2-1.1) | 0.093 | 0.6(0.1-94.6) | 0.603 |
| *AHR**:adjusted by age, gender, marital status, baseline BMI, baseline CD4 cell count, and clinical disease(including TB effection, Skin lesion, Thrush, Oral hairy leukoplakia, Persistent diarrhea, Continuous or intermittent fever, Recurrent severe bacterial infections, Disseminated non-tuberculosis bacillus infection, Oesophageal candidiasis, Extrapulmonary cryptococcus infection, Yersinia pneumocystis pneumonia, Disseminated fungal disease, Cytomegalovirus infection, Extra pulmonary tuberculosis, Repeated severe bacterial pneumonia, Chronic herpes simplex virus infection, Herpes zoster, Toxoplasma encephalopathy, Brain lymphoma, WHO clinical stage). | | | | | | |
